# Early application of modified A-OK protocol for amniotic fluid embolism: Case series report

**DOI:** 10.1097/MD.0000000000045462

**Published:** 2025-10-24

**Authors:** Shanshan He, Bo Tao

**Affiliations:** a Department of Anesthesiology, West China Second University Hospital, Sichuan University, Chengdu, China; b Department of Radiology, West China Hospital of Sichuan University, Chengdu, China.

**Keywords:** amniotic fluid embolism, atropine, disseminated intravascular coagulation, ketorolac, ondansetron

## Abstract

**Rationale:**

Amniotic fluid embolism (AFE) is a rare but catastrophic obstetric emergency with high mortality, characterized by sudden cardiovascular collapse, hypoxia, and coagulopathy. Current management is primarily supportive, but emerging case reports suggest a potential benefit from a novel regimen combining atropine, ondansetron, and ketorolac (A-OK). However, due to AFE’s extreme rarity, large-scale studies are lacking.

**Patient concerns:**

Three women with suspected AFE presented with acute hemodynamic instability, hypoxia, or coagulopathy during or shortly after delivery.

**Diagnoses:**

AFE was clinically diagnosed based on sudden cardiovascular collapse, respiratory distress, and coagulopathy in the peripartum setting.

**Interventions:**

A modified A-OK protocol, atropine, granisetron (replacing ondansetron), and lornoxicam (replacing ketorolac) (A-GL), was administered early alongside standard resuscitation measures.

**Outcomes:**

Rapid hemodynamic stabilization and improved oxygenation were observed within minutes of A-GL administration. All 3 patients survived with favorable outcomes.

**Lessons:**

These cases suggest that early A-OK-based therapy (or its modified A-GL regimen) may improve outcomes in AFE. Further research is warranted to validate this approach and refine treatment protocols.

## 1. Introduction

Amniotic fluid embolism (AFE) is hypothesized to occur when amniotic fluid enters the maternal circulation, triggering a cascade of inflammatory responses that result in pulmonary vasoconstriction, pulmonary hypertension, severe hypoxemia, and ultimately cardiovascular collapse. Additionally, amniotic fluid is thought to activate the extrinsic coagulation pathway, leading to consumptive coagulopathy and disseminated intravascular coagulation (DIC).^[1]^ Current management of AFE includes interventions that attempt to counteract these pathologic processes.^[2]^ The use of atropine (1 mg IV), ondansetron (8 mg IV), and ketorolac (30 mg IV) (A-OK) is a widely discussed new adjunctive treatment for AFE.^[3]^ However, documented cases demonstrating A-OK’s efficacy remain scarce. At our institution, granisetron and lornoxicam have replaced ondansetron and ketorolac, respectively (A-GL). We present 3 cases from the past 2 years in which early administration of this modified A-OK regimen successfully treated suspected AFE, with follow-up revealing no maternal or neonatal complications.

## 2. Detailed case description

Case 1: A 33-year-old woman, G5P1 + 3 at 39 1/7 weeks of gestation, presented for emergency cesarean section due to Grade III meconium-stained amniotic fluid. Combined spinal-epidural anesthesia was successfully performed, with an analgesia block level at T6. Before skin incision, the patient complained of chest tightness and suddenly her blood pressure decreased to 57/38 mm Hg, and oxygen saturation dropped to 44% on room air. Ephedrine (6 mg IV) and epinephrine (20 μg IV) were administered, and then rapid sequence intubation was performed with 8 mg of etomidate and 100 mg of succinylcholine. Blood pressure at that time was 114/72 mm Hg and SpO2 increased to 84% with mechanical ventilation. Two minutes later, a newborn was delivered with 1–5–10 minute Apgar scores of 7–8–9. Three minutes after delivery, the patient’s blood pressure further dropped to 44/24 mm Hg without notable hemorrhage, her heart rate increased to 160 bpm, and SpO₂ remained 83% under mechanical ventilation with 100% oxygen. AFE was highly suspected by the anesthesiologist. Atropine (0.5 mg IV), granisetron (3 mg IV), and lornoxicam (8 mg IV) were administered. In addition, a second dose of epinephrine (20 μg IV) was given intravenously. Within 1 minute, the patient’s SpO₂ recovered to 97% and blood pressure increased to 100/31 mm Hg. Subsequently, norepinephrine was administered at a rate of 0.2 μg/kg/min to maintain blood pressure. Hydrocortisone (100 mg IV) and dexamethasone (10 mg IV) were then administered. Bedside echocardiography showed normal cardiac chamber sizes and left ventricular systolic function. Thirty-three minutes after delivery, the patient’s laboratory results (blood samples collected before surgery) showed thrombocytopenia (platelet count: 50 × 10⁹/L). At this point, estimated blood loss exceeded 1000 mL, with signs of ongoing active bleeding and hemodynamic instability. Tranexamic acid was immediately administered as well as blood products, which included fibrinogen concentrate, cryoprecipitate, and platelets. Twenty-eight minutes later, coagulation results showed prolonged prothrombin time (PT) and activated partial thromboplastin time (aPTT), and hypofibrinogenemia (**Table 1**). Fresh frozen plasma (FFP) and prothrombin complex concentrate were additionally administered. Finally, she received 3U of red blood cells, 1000 mL of FFP, 6U of platelets, 10 g of fibrinogen, 600IU of prothrombin complex concentrate, and 10U of cryoprecipitate. The patient and newborn had an uneventful postoperative recovery and were discharged on postoperative day 11.

**Table 1:** Laboratory values indicating disseminated intravascular coagulation (DIC) in Case 1.

Variable	Reference rang	Before surgery	Onset of symptoms	Transferred from OR
Hematocrit (%)	35–45	41.2	48.8	31.8
Hemoglobin (g/L)	110–150	139	164	106
White-cell count (10^9^/L)	3.5–9.5	9.1	9.6	7.6
Platelet count (10^9^/L)	100–450	214	50	93
Red-cell count (10^12^/L)	3.8–5.1	4.53	5.27	3.42
Prothrombin time (PT, sec)	8.0–14.0	11.8	20.8	15.5
Activated partial thromboplastin time (aPTT, sec)	17.7–37.7	24.0	127.5	49.9
Fibrinogen (mg/dL)	200–400	537	<50	100

Case 2: A 35-year-old woman gravida 2, para 2013 presented to our hospital at 37 + 1 weeks of gestation for induction of labor due to gestational diabetes, gestational hypertension, and low-lying placenta. She presented for cervical ripening at 10:50 am. At 7 pm, the patient experienced uterine contractions with 5–6 minutes intervals, accompanied by retching, restlessness, and mild chest tightness. The cervical exam showed 1 + cm cervical dilatation, 90% effacement, and some small sludged blood. The patient suddenly developed cyanosis with oxygen saturation of 75% to 81%, but other vital signs were normal. The differential diagnosis at that time included AFE versus massive pulmonary embolism (PE). Then she was quickly moved to the operating room. Several nurses, anesthesia staff, and obstetric physicians quickly assembled for an emergency cesarean section. Other members of the multidisciplinary team were also requested to participate in the rescue efforts. Upon arriving in the operating room, the patient’s oxygen saturation dropped to 67%, blood pressure decreased to 74/37 mm Hg, and the patient exhibited extreme agitation and complained of a feeling of impending doom. The anesthesia team initiated the A-GL protocol within 1 minute after excluding a history of venous thromboembolism. As the patient responded to medical management, her blood pressure and oxygen saturation improved within 2 minutes. The patient quickly underwent emergency cesarean section under general endotracheal intubation anesthesia with rapid sequence intubation. One minute after general anesthesia induction, the patient delivered an infant with Apgar scores of 8, 9, and 10 at 1, 5, and 10 minutes, respectively, with minimal bleeding. No embolus was noted during echocardiogram in the proximal pulmonary arteries, and PE could be excluded based on medical history. Ten minutes after delivery, maternal blood pressure dropped significantly again without notable hemorrhage. At the same time, the obstetrician noted signs of DIC at the cesarean incision site. AFE could be clinically diagnosed. Norepinephrine was continuously infused to maintain hemodynamic stability, and 4 g fibrinogen infusion was initiated. Initial laboratory results suggested DIC (Table 2), consistent with our hypothesis. Massive transfusion protocol was initiated to correct the coagulation dysfunction. Other AFE protocol measures were also immediately activated, including: inhaled nitric oxide, continuous norepinephrine and milrinone infusion for hemodynamic support, as well as administration of calcium gluconate, uterotonics, tranexamic acid, and methylprednisolone 80 mg. An emergency hysterectomy was decisively performed after multidisciplinary discussion due to uncontrolled bleeding and refractory hemodynamic instability. During the procedure, the estimated blood loss was 2,530 mL. She ultimately received a total of 14 g fibrinogen, 2 units of Rh-negative red blood cells, 1,000 mL of FFP, 6 units of cryoprecipitate, and 2750 mL fluid over a 3-hour period. A special feature of this case was that the patient had AB Rh-negative blood type but received B Rh-positive plasma and AB Rh-positive cryoprecipitate. Fortunately, no adverse transfusion reactions occurred during this process. She was transferred to the intensive care unit intubated but without vasopressor support, and was extubated on postoperative day 1 and discharged on day 6 with intact neurological outcomes.

**Table 2:** Laboratory values indicating disseminated intravascular coagulation (DIC) in Case 2.

Variable	Reference range	Before surgery	Onset of symptoms	Transferred from OR
Hematocrit (%)	35–45	39.9	41.6	22.2
Hemoglobin (g/L)	110–150	133	133	75
White-cell count (10^9^/L)	3.5–9.5	10.3	10.6	17.5
Platelet count (10^9^/L)	100–450	186	23	109
Red-cell count (10^12^/L)	3.8–5.1	4.59	4.59	2.52
Prothrombin time (PT, sec)	8.0–14.0	11.5	20	15.4
Activated partial thromboplastin time (aPTT, sec)	17.7–37.7	25.0	98.7	29.1
Fibrinogen (mg/dL)	200–400	496	43	224

Case 3: A 31-year-old woman, G2P1 at 39 + 2 weeks of gestation, underwent epidural labor analgesia after developing regular uterine contractions with cervical dilation of 1 cm. Approximately 3 hours later, full cervical dilation was achieved. The patient complained of chest discomfort, which was relieved after being given oxygen. Subsequently, fetal heart rate tracing showed a few late decelerations that could not be restored with intrauterine resuscitation. An episiotomy-assisted forceps delivery was promptly performed. The patient delivered an infant with Apgar scores of 10, 10, and 10 at 1, 5, and 10 minutes, respectively. To treat uterine atony and intraoperative hemorrhage, the patient received oxytocin (100 µg IV), tranexamic acid (1 g IV), and calcium gluconate (1 g IV) after delivery. She was noted to have signs of DIC during perineal wound suturing, with bleeding at that time of only 200 mL. The patient again complained of chest discomfort and obvious chills with a body temperature of 38°C. Although no hypotension or hypoxemia was noted, atypical amniotic fluid embolism was highly suspected by the obstetric team. A multidisciplinary resuscitation team was immediately activated. Ultrasound for deep vein thrombosis was negative, echocardiography revealed only mild tricuspid regurgitation, and lung ultrasound showed no abnormalities. Initial laboratory findings were consistent with DIC (Table 3), although there was no notable hemorrhage. Blood products were transfused to correct the deficits appropriately, while the A-GL medications (atropine, granisetron, and lornoxicam) were also administered. The patient eventually received a total of 6 units of packed red blood cells, 750 mL of FFP, 2 units of cryoprecipitate, and 8 g of fibrinogen over a 3-hour period. The patient did not experience decreased blood pressure or oxygen saturation throughout the entire rescue process. The patient was discharged home on postoperative day 4.

**Table 3:** Laboratory values indicating disseminated intravascular coagulation (DIC) in Case 3.

Variable	Reference range	Before surgery	Onset of symptoms	Transferred from OR
Hematocrit (%)	35–45	34.9	28	31.8
Hemoglobin (g/L)	110–150	114	92	108
White-cell count (10^9^/L)	3.5–9.5	8.3	9.4	12.9
Platelet count (10^9^/L)	100–450	166	78	81
Red-cell count (10^12^/L)	3.8–5.1	4.02	3.24	3.69
Prothrombin time (PT, sec)	8.0–14.0	12.1	21.2	14.6
Activated partial thromboplastin time (aPTT, sec)	17.7–37.7	28.2	66.6	33.8
Fibrinogen (mg/dL)	200–400	454	<50	265

Ethical approval for this case report was waived by the Ethics Committee of West China Second University Hospital of Sichuan University, as it was a retrospective analysis of anonymized clinical data from routine medical care.

## 3. Discussion

AFE is a rare, unpredictable, and potentially lethal complication of childbirth. It can lead to severe maternal and fetal disabilities or even death. Early recognition and initiation of treatment are crucial to increase the patient’s chances of survival. However, this can be challenging, as AFE is a clinical diagnosis of exclusion that requires ruling out other conditions, including massive hemorrhage, infectious shock, anesthetic accidents, allergic reactions, and PE. Many early signs and symptoms are nonspecific. The first step is to be alert to the risk factors for AFE, which include placenta previa, preeclampsia, cesarean section, forceps delivery, maternal age > 35 years, and vacuum delivery. Other early, often overlooked, nonspecific prodromal symptoms include hypotension, foaming at the mouth, abnormal fetal heart rate, loss of consciousness, bleeding, changes in uterine tone, and seizures. Severe fetal bradycardia can be the first manifestation of AFE, as seen in our third case. The classic triad of AFE typically appears within 2 hours before fetal delivery and 30 minutes after placental expulsion, consisting of hypoxia, hypotension, and coagulopathy. Atypical AFE may present only with nonspecific symptoms and DIC. All 3 of our patients had at least one risk factor and presented classically with a preceding aura. Our 3 patients met the clinical diagnostic criteria for AFE created by Erez.^[4]^ Furthermore, echocardiography, which shows right ventricular dysfunction, helps diagnose AFE and is associated with the occurrence of cardiac arrest, while also helping to rule out PE.^[5]^

The current recommendations from the Society for Maternal-Fetal Medicine suggest the use of sildenafil, dobutamine, milrinone, inhaled nitric oxide, prostacyclin, and norepinephrine when managing AFE.^[2]^ However, the mechanism of action of these agents is supportive treatment and does not address the potential underlying mechanism of AFE. Our treatment approach also followed the guidelines of the Maternal-Fetal Medicine Association. They received respiratory and circulatory support as well as extensive blood product transfusions. The difference from the recommendations is that we administered A-GL in the early stages for these 3 cases. The patients responded to this treatment, with rapid improvement in blood pressure and oxygen saturation within minutes after A-GL administration, although vasopressor agents such as norepinephrine also contributed to stabilizing hemodynamics. Additionally, a few case reports have suggested the use of extracorporeal membrane oxygenation to treat severe cardiovascular failure caused by AFE_._^[6]^

The traditional view holds that AFE occurs when components of amniotic fluid enter the maternal circulation, obstructing pulmonary capillaries, leading to pulmonary arterial flow obstruction, and subsequently causing hypoxemia, pulmonary hypertension, right heart failure, and even death. This is known as the mechanical obstruction theory of AFE. However, further research into the pathophysiological mechanisms of AFE has revealed that vagus nerve activation, along with the synergistic effects of serotonin and thromboxane, causes pulmonary vasoconstriction, which is the primary mechanism underlying respiratory and circulatory failure. Additionally, the presence of amniotic fluid and thromboxane activates the extrinsic pathway of the coagulation cascade, leading to consumptive coagulopathy and ultimately resulting in DIC.^[1,3]^ Based on this mechanism, new therapies have shown potential. It has been reported that antiserotonin, antithromboxane, and vagolytic therapy were the mechanisms for the resuscitation of a patient with AFE.^[3]^ Atropine can block vagal stimulation, improving cardiovascular function rather than simply providing cardiovascular support. Ondansetron is believed to block serotonin, while ketorolac could block coagulation cascade activation by inhibiting thromboxane. If ondansetron is unavailable, any 5-HT3 receptor antagonist can serve as a substitute, and if ketorolac is unavailable, any nonsteroidal anti-inflammatory drug can be used as an alternative. Some case reports have suggested that combination therapy of traditional supportive measures and A-OK may improve survival.^[3,7]^

The extremely low incidence of amniotic fluid embolism makes its management highly challenging. Regular simulations are essential for preparing for this rare and often fatal complication. The involvement of a multidisciplinary team and rapid recognition of the diagnosis with appropriate treatment were critical to the patients’ survival. At our institution, we routinely conduct AFE simulation drills, with particular emphasis on the early application of A-OK therapy, which enabled us to respond promptly when patients presented with suspected AFE. We believe our patients survived with no severe long-term consequences normally associated with this frightening syndrome because of rapid diagnosis and use of these protocolized interventions.

## 4. Conclusion

Our cases demonstrate that early A-OK protocol implementation, combined with AFE protocolized resuscitation, and the involvement of multidisciplinary team contributed to favorable maternal and neonatal outcomes without neurological sequelae. However, as AFE remains an exclusionary clinical diagnosis, further research and additional case reports were needed to clarify the optimal timing of A-OK administration and address potential risks associated with diagnostic uncertainty.

## Author contributions

**Conceptualization:** Shanshan He, Bo Tao.

**Data curation:** Shanshan He.

**Formal analysis:** Shanshan He.

**Writing – original draft:** Shanshan He.

**Writing – review & editing:** Bo Tao.
